# Potential impact of infant feeding recommendations on mortality and HIV-infection in children born to HIV-infected mothers in Africa: a simulation

**DOI:** 10.1186/1471-2334-8-66

**Published:** 2008-05-16

**Authors:** Julius Atashili, Linda Kalilani, Vidyunmala Seksaria, Emily E Sickbert-Bennett

**Affiliations:** 1 Department of Epidemiology, University of North Carolina, Chapel Hill, USA; 2 Center for the Study and Control of Communicable Diseases, Yaoundé, Cameroon

## Abstract

**Background:**

Although breast-feeding accounts for 15–20% of mother-to-child transmission (MTCT) of HIV, it is not prohibited in some developing countries because of the higher mortality associated with not breast-feeding. We assessed the potential impact, on HIV infection and infant mortality, of a recommendation for shorter durations of exclusive breast-feeding (EBF) and poor compliance to these recommendations.

**Methods:**

We developed a deterministic mathematical model using primarily parameters from published studies conducted in Uganda or Kenya and took into account non-compliance resulting in mixed-feeding practices. Outcomes included the number of children HIV-infected and/or dead (cumulative mortality) at 2 years following each of 6 scenarios of infant-feeding recommendations in children born to HIV-infected women: Exclusive replacement-feeding (ERF) with 100% compliance, EBF for 6 months with 100% compliance, EBF for 4 months with 100% compliance, ERF with 70% compliance, EBF for 6 months with 85% compliance, EBF for 4 months with 85% compliance

**Results:**

In the base model, reducing the duration of EBF from 6 to 4 months reduced HIV infection by 11.8% while increasing mortality by 0.4%. Mixed-feeding in 15% of the infants increased HIV infection and mortality respectively by 2.1% and 0.5% when EBF for 6 months was recommended; and by 1.7% and 0.3% when EBF for 4 months was recommended. In sensitivity analysis, recommending EBF resulted in the least cumulative mortality when the a) mortality in replacement-fed infants was greater than 50 per 1000 person-years, b) rate of infection in exclusively breast-fed infants was less than 2 per 1000 breast-fed infants per week, c) rate of progression from HIV to AIDS was less than 15 per 1000 infected infants per week, or d) mortality due to HIV/AIDS was less than 200 per 1000 infants with HIV/AIDS per year.

**Conclusion:**

Recommending shorter durations of breast-feeding in infants born to HIV-infected women in these settings may substantially reduce infant HIV infection but not mortality. When EBF for shorter durations is recommended, lower mortality could be achieved by a simultaneous reduction in the rate of progression from HIV to AIDS and or HIV/AIDS mortality, achievable by the use of HAART in infants.

## Background

An estimated 2.3 million children under 15 years were living with human immunodeficiency virus (HIV) infection, and 700,000 children were newly infected in 2005 alone [1]. Ninety percent of these HIV infections were acquired through mother-to-child-transmission (MTCT). Vertical transmission of the HIV virus from mother to child can occur during pregnancy, delivery or postnatal through breast-milk [2]. Rates of MTCT range from 5–25% in developed and 13–42% in developing countries[3]. Data from various studies indicate that breast-feeding may be responsible for one-third to one-half of HIV infections in infants and young children in Africa[2].

The reduction of HIV transmission during lactation is one of the most pressing global health dilemmas confronting health policy makers and HIV-infected women in many regions of the world [4-6]. Replacement-feeding prevents breast-milk transmission of HIV. However, in resource-limited settings, access to replacement-feeding is hindered by costs, poor water quality and sanitation, cultural practices and stigma associated with not breast-feeding [7-9]. In addition, the protection offered by breast-feeding against diarrheal and respiratory diseases which cause high infant mortality rates, needs to be weighed against the risk of transmitting HIV.

It has long been recommended that women who are HIV positive should avoid breast-feeding and use replacement-feeding when it is acceptable, feasible, affordable, sustainable and safe (AFASS) [10]. In cases were this is not possible, exclusive breast-feeding is recommended for the first months of life, followed by rapid weaning as soon as it is feasible, depending on the individual woman's situation, and taking into account the possible increased risk of HIV transmission with mixed-feeding during the transition period between exclusive breast-feeding and complete cessation of breast-feeding.

Several researchers have modeled the risks and benefits of replacement versus breast-feeding for HIV-infected mothers in developing countries [6,7,11-18]. However, these modeling studies primarily examined the impact of exclusive breast-feeding versus replacement-feeding with little attention to the recommended duration of exclusive breast-feeding or the impact of poor compliance to these recommendations.

Taking these limitations into consideration, we developed a model that examined the potential impact of different infant-feeding recommendations on the overall mortality, burden of HIV and AIDS in children less than 2 years of age, and also examined the impact of varying the duration of breast-feeding and the rate of compliance to infant-feeding recommendations. We chose *a priori *to derive parameter sources for this model from Uganda and Kenya, two East African countries where the epidemiology is relatively well documented. In addition, we assessed the impact of variations to the chosen parameters through a sensitivity analysis. In contrast to previous models of time-to-death as a single outcome, we chose to model both cumulative mortality and infection proportions at 2 years. Our choice of these two outcome measures was designed to address the fact that communities may be as concerned with the number of children living with HIV/AIDS after a certain time period as they could be about the number of children dead. Further, cumulative proportions of children living with HIV/AIDS or dead represent statistics that are easy-to-interpret and understand and thus are at least as useful as time-to-event statistics (hazard or rates).

## Methods

### Model characteristics

**Figure 1:**
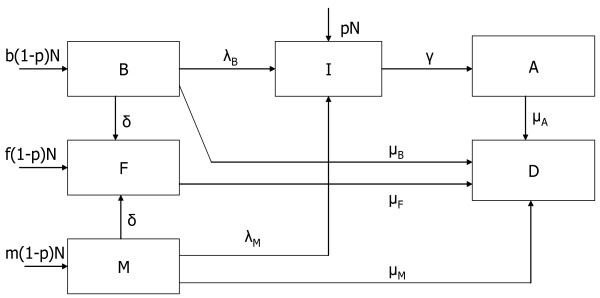
Model compartment and parameters.

We developed a compartmental, deterministic model to simulate the effects of different breast-feeding recommendations in HIV-infected women in a typical sub-Saharan setting (Figure 1). This type of model was chosen for its simplicity and the direct interpretation of results. This model simulated a population of N children born to women who were HIV-positive during pregnancy. A proportion (p) of these children were infected at birth, whilst (1-p), of the N children were born HIV-negative. Though, in practice, infants' HIV status is usually based on a PCR test at 6 weeks, we assumed that infants who tested positive at 6 weeks were positive right from birth. Infants born infected with HIV (I), can later progress to develop AIDS (A). Non-infected infants were distributed according to their feeding mode into the following three compartments; exclusively breast-fed (B), mixed-fed (M), and replacement-fed (F), representing the proportions b, m, f respectively. In our model, exclusive breast-feeding was defined as feeding the infant breast-milk only, mixed-feeding was defined as feeding the infant breast-milk and other non-breast-milk liquids, and replacement-feeding meant that the infant was not given breast-milk but other non-breast-milk liquids. Non-infected infants could eventually get HIV-infected (I), and HIV-infected infants could progress to AIDS (A). Death could result from AIDS and AIDS-related causes or from non-AIDS related causes.

The model assumed that post-natal HIV transmission occurred solely through breast-feeding with no difference in the HIV transmission rates by gender. Exclusively breast-fed infants were infected with HIV at a rate of λ_B _while infants receiving mixed-feeding were infected at a rate of λ_M_. Evidence for higher HIV transmission in infants receiving mixed-feeding when compared to exclusively breast-fed infants[19] was taken into account by using values of λ_M _that were higher than λ_B_. We assumed that pre- and intra-partum use of antiretroviral therapy (ART) by the mother did not have any effect on the cumulative 2-year postpartum risk of HIV transmission through breast-feeding. The base model assumed no use of ART in the postpartum period (as this is not a common practice in many developing countries)[10]. This assumption however was relaxed in the sensitivity analysis by varying λ_M _and λ_B_.

In addition, we assumed that the risk of HIV transmission in breast milk is constant[20]. By using an average value for transmission risk, we underestimate the rate in periods of truly high transmission, but this is compensated by overestimating the estimates in periods of truly low transmission such that outcome measures of cumulative proportions remain valid estimates. HIV-infected infants progressed to AIDS at a rate γ, estimated from the inverse of the average duration from HIV infection to the onset of AIDS in infants. The model assumed that progression from HIV to AIDS did not depend on the mode of feeding. Infants with AIDS died at a rate of μ_A_. We assumed that the mortality in HIV-infected children was mainly due to AIDS-related illness (with non-AIDS related mortality being negligible).

Weaning in exclusively breast-fed infants and infants receiving mixed-feeding occurred at a rate δ; for this simulation, weaning was assumed to be abrupt. This rate was estimated from the inverse of the average duration of breast-feeding in the population. Mortality rates in uninfected infants depended on the mode of feeding with infants receiving exclusively breast-milk, no breast-milk and mixed-feeding dying at rates of μ_B_, μ_F_, and μ_M_, respectively.

The following differential equations, with a time step of one week, were used to model the weekly rate at which infants moved in and out of compartments. B(t), F(t), M(t), I(t), and A(t), represented the number of infants respectively in compartments B, F, M, I and A at time t.

(1) dB/dt = b(1-p)N - [λ_B _+ δ + μ_B_]B(t)

(2) dF/dt = f(1-p)N + δ[B(t) + M(t)] - μ_F_F(t)

(3) dM/dt = m(1-p)N - [λ_M _+ δ + μ_M_]M(t)

(4) dI/dt = pN + λ_B_B(t) + λ_M_M(t) - γI(t)

(5) dA/dt = γI(t) - μ_A_A(t)

(6) dD/dt = μ_A_A(t) + μ_B_B(t) + μ_F_F(t) + μ_M_M(t)

Six unique infant-feeding scenarios were analyzed (Table 1): these were defined by the recommended mode and duration of feeding as well as compliance to the breast-feeding recommendation in the population of HIV-infected women. Breast-feeding could be prohibited in all infants (scenarios U and X), or breast-feeding could be recommended for a duration of 6 months (scenarios V and Y) or for a duration of 4 months (scenarios W and Z). Three of these scenarios are idealistic, (U, V, W) assuming complete (100%) compliance while the other three scenarios (X, Y, Z) were more realistic assuming 85% compliance in exclusively breast-fed infants and 70% compliance in infants not breast-fed [21-23].

**Table 1:** Characteristics of scenarios considered in modeling the potential impact of different breast-feeding recommendations in children born to HIV-infected women in sub-Saharan Africa

	**Idealistic scenarios**			Realistic* scenarios		
**Recommendation**	**All infants not breast-fed (U)**	**All infants exclusively breast-fed for 6 months (V)**	**All infants exclusively breast-fed for 4 months (W)**	**All infants not breast-fed (X)**	**All infants exclusively breast-fed for 6 months (Y)**	**All infants exclusively breast-fed for 4 months (Z)**

**Proportion Not breast-fed (f)**	1.00	0.00	0.00	0.70	0.00	0.00
**Proportion exclusively breast-fed (b)**	0.00	1.00	1.00	0.00	0.85	0.85
**Proportion on mixed-feeding (m)**	0.00	0.00	0.00	0.30	0.15	0.15
**Recommended breast-feeding duration (1/δ months)**	NA	6	4	NA	6	4

* Realistic scenarios assume 85% compliance in exclusively breast-fed infants and 70% compliance in replacement-fed infants [21–23].

The primary model outcomes were: the cumulative mortality at 2 years (104 weeks) defined as *D*_*t *= 104_/*N*, the proportion of children infected with HIV at 2 years defined as (*I*_*t *= 104 _+ *A*_*t *= 104_)/*N*, and the proportion of children either living with HIV/AIDS or dead at 2 years defined as *D*_*t *= 104 _+ *I*_*t *= 104 _+ *A*_*t *= 104_/*N*. It is worth noting that the latter combined measure counts each infant only once (not twice). Because this is a compartmental model, at any given time each infant is in one and only one compartment. So at time 2-year, infants who died from HIV will be in the D compartment, not in the I compartment. The assessment endpoint was set a priori at 2 years with the assumption that infant-feeding patterns negligibly affected child mortality after 2 years.

### Parameter estimates

Estimates of parameters used in the model were obtained from published articles from a Medline search using the MeSH keywords "Infant + Feeding + HIV" as well as guideline documents published by the World Health Organization publication (WHO). Because of the heterogeneity in parameter values across regions, the parameters were chosen primarily from studies conducted in Kenya or Uganda. For estimates that were not documented in these two countries we used projections from other countries in the region (with similar epidemiology). The specific values used for each parameter are shown in Table 2.

**Table 2:** Definition, values and sources of parameters used in modeling the potential impact of different breast-feeding recommendations in children born to HIV-infected women in sub-Saharan Africa

**Parameter**	**Definition**	**Base model**	**Source of estimates**
N	Population size of children born to HIV-infected women	100000	-
P	Proportion of infants born HIV positive	0.08	[25]
λ_B_	Rate of HIV infection from exclusive breast-feeding (cases/person-week)	0.0019	[21, 23]
λ_M_	Rate of HIV infection from mixed-feeding (cases/person-week)	0.0027	[26]*
Γ	Rate of progression from HIV to AIDS= (average duration of non-AIDS HIV infection)^-1^	0.016	[27]
μ_M_	Mortality rate in mixed-fed infants (per person-week)	0.00087	**
μ_F_	Mortality rate in replacement-fed infants (per person-week)	0.00096	[28]
μ_B_	Mortality rate in exclusively breast-fed infants (per person-week)	0.00078	[28]
μ_A_	Mortality rate due to HIV/AIDS (per person-week)	0.005	[28]
B	Proportion of uninfected infants at birth that are exclusively breast-fed	†	-
F	Proportion of uninfected infants at birth that are exclusively replacement-fed	†	-
M	Proportion of uninfected infants at birth that receive mixed feeding	†	-

* In the absence of specific estimates in Uganda/Kenya, used this study to estimate that transmission rate in mixed fed is 40% higher than that in exclusively breast-fed.

** In the absence of data: mortality in mixed-fed infants was assumed to be the mean of the mortality in exclusively breast-fed and replacement-fed infants.

† Values varied by scenario (see Table 1)

### Sensitivity analysis

Sensitivity analyses were conducted to evaluate the potential impact of the choice of parameters. We performed univariate sensitivity analyses, varying one parameter while holding the rest of the parameters in the model constant. The following parameters were varied: the recommended duration of breast-feeding from 1 to 6 months; compliance to the recommended infant-feeding from 10 to 100%, the proportion of infants born HIV-infected from 1% to 25%; the mortality rates in breast-fed, mixed-fed and replacement-fed infants from 0 to 200 per thousand children, taking into account differences in access to clean water and health care facilities according to geographical settings. Parameters that could be influenced by the greater availability of anti-retroviral therapy such as the rate of infection in breast-fed infants, the rate of progression from HIV to AIDS and the mortality rate due to HIV/AIDS were also varied.

## Results

### Base model

**Figure 2:**
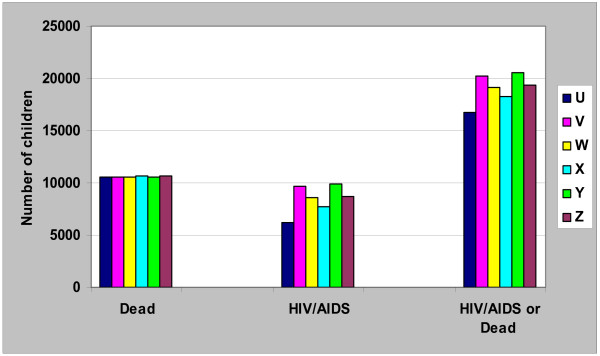
**Base model of six infant-feeding scenarios in 100,000 infants born to HIV+ mothers: Number of Children with HIV/AIDS, dead, or both at 2 years**. U: Exclusive replacement-feeding (ERF) with 100% compliance; V: EBF for 6 months with 100% compliance; W: EBF for 4 months with 100% compliance: X: ERF with 70% compliance; Y: EBF for 6 months with 85% compliance; Z: EBF for 4 months with 85% compliance.

In the scenarios with 100% compliance (scenarios U, V, W) to the infant-feeding recommendations, exclusive replacement-feeding (scenario U) resulted in the least number of children with HIV/AIDS at 2 years (Figure 2). The proportion of children with HIV/AIDS at 2 years was 6.2%, 8.6% and 9.7% for replacement-fed, and infants breast-fed for 4 or 6 months respectively. However, the cumulative mortality at 2 years was very similar for each of the three scenarios: 10.55% in infants who had replacement-feeding compared to 10.57% in infants who were breast-fed for 4 months and 10.53% for infants breast-fed for 6 months. Considering all the outcomes, HIV/AIDS and mortality at 2 years together, replacement-feeding was the best feeding option if there was 100% compliance (scenario U, Figure 2) as it resulted in the least number of children affected by HIV/AIDS or death, while breast-feeding for 6 months (scenario V) had the highest combined morbidity/mortality.

Taking into account the limited compliance to recommendations and assuming 70% compliance when replacement-feeding was recommended (scenario X), and 85% compliance when exclusive breast-feeding (for 4 (scenario Z) or 6 months (scenario Y)) was recommended, the number of infants infected with HIV, and having AIDS at 2 years was still lower with replacement-feeding compared to exclusive breast-feeding for 4 or 6 months. However, compared to the scenario where there was 100% compliance, the number of children with HIV/AIDS at 2 years increased by 24% for replacement-feeding with 70% compliance (scenario U vs. X). By contrast, the number of children with HIV/AIDS at 2 years increased only by 1.7% (scenario W vs. Z) and 2.1% (scenario V vs. Y), for infants who were exclusively breast-fed for 4 and 6 months respectively, when compliance was 85% versus 100%. The cumulative mortality increased only by 0.9%, 0.3% and 0.5% in children who had replacement-feeding (scenario U vs. X) or were breast-fed for 4 (scenario W vs. Z) and 6 months (scenario V vs. Y) respectively when compliance was reduced from 100% to 70% for replacement-feeding and 85% for exclusive breast-feeding. When limited compliance was taken into account, the least total number of children with HIV/AIDS or dead at 2 years was obtained when replacement-feeding was recommended (scenario X), followed by exclusive breast-feeding for 4 months (scenario Z) and lastly, exclusive breast-feeding for 6 months(scenario Y) (Figure 2).

### Sensitivity analysis

#### Duration of breast-feeding

**Figure 3:**
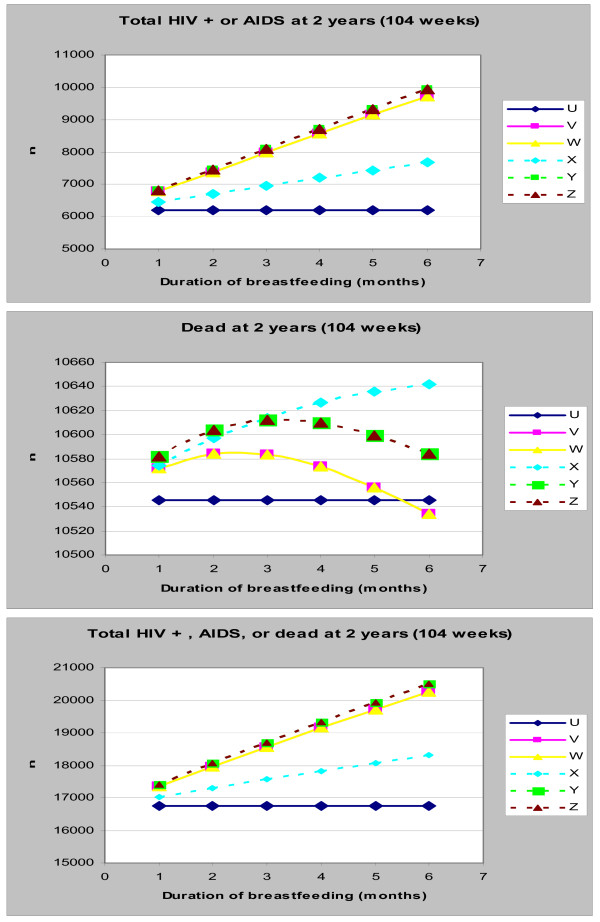
**(a-c): Impact of Duration of Breast-feeding on the Number of Children with HIV/AIDS (top graph), dead (middle graph) or both (bottom graph) at 2 years**. U: Exclusive replacement-feeding (ERF) with 100% compliance; V: EBF for 6 months with 100% compliance; W: EBF for 4 months with 100% compliance: X: ERF with 70% compliance; Y: EBF for 6 months with 85% compliance; Z: EBF for 4 months with 85% compliance.

With 100% compliance, increasing the recommended duration of breast-feeding resulted in an increase in the number of children infected with HIV/AIDS (Figure 3a). When compliance was 70% for the replacement-feeding recommendation (scenario X), the number of children with HIV/AIDS also increased with any increase in the recommended duration of breast-feeding.

Varying the recommended duration of breast-feeding had very little impact on the cumulative mortality – between 10.5% and 10.7% of all infants were dead at 2 years irrespective of scenario or duration (Figure 3b). Despite this limited overall impact, increasing the recommended duration of breast-feeding from 1 to 2 months resulted in an initial increase in 2-year cumulative mortality. The maximum mortality was attained at a recommended duration between 2 and 3 months, followed by a progressive decline in cumulative mortality as the duration increased to 6 months. Compared to replacement-feeding, slightly fewer infants were dead at 2 years only when exclusive breast-feeding for more than 5 months was recommended.

With a reduced compliance of 70%, cumulative mortality increased in infants who had replacement-feeding (scenario X) with increased recommended duration of breast-feeding (Figure 3b). By contrast, a reduced compliance of 85% following a recommendation of exclusive breast-feeding (scenario Y, Z) resulted in an initial increase in cumulative mortality, with a maximum being attained when breast-feeding was recommended for 3–4 months (Figure 3b). Furthermore, recommending breast-feeding for 3 months or more (with reduced compliance) (scenario Y, Z) resulted in fewer deaths than recommending replacement-feeding with reduced compliance (scenario X).

#### Compliance to recommended infant-feeding method

**Figure 4:**
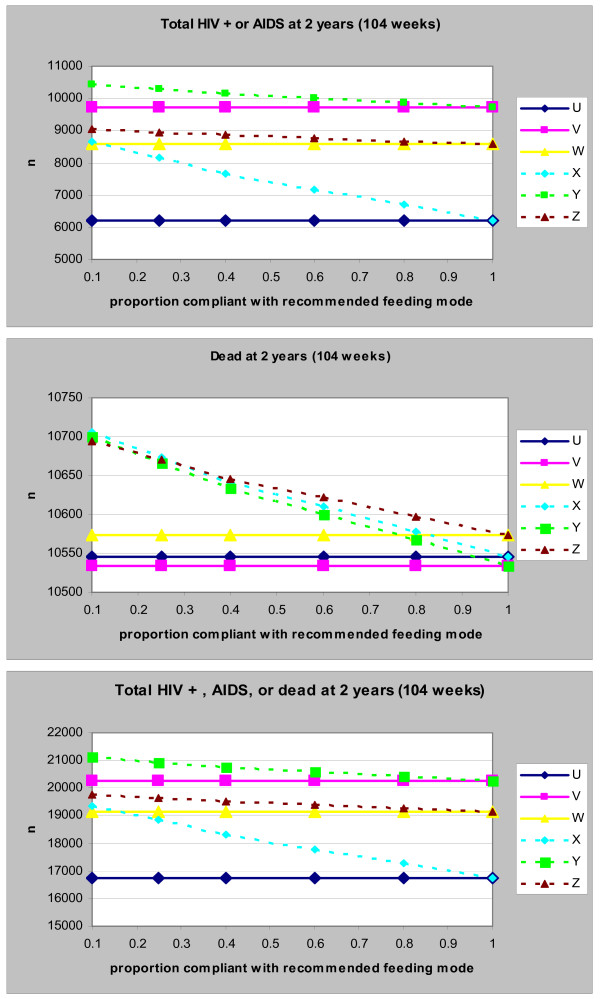
**(a-c): Impact of Compliance to Feeding Recommendations on the Number of Children with HIV/AIDS (top graph), dead (middle graph) or both (bottom graph) at 2 years**. U: Exclusive replacement-feeding (ERF) with 100% compliance; V: EBF for 6 months with 100% compliance; W: EBF for 4 months with 100% compliance: X: ERF with 70% compliance; Y: EBF for 6 months with 85% compliance; Z: EBF for 4 months with 85% compliance.

For all scenarios, decreasing the compliance to recommended infant-feeding methods resulted in an increase in the number of children infected with HIV and or dead at 2 years (Figure 4c). The impact of compliance on HIV infection and/or cumulative mortality was highest with replacement-feeding than it was with breast-feeding. Though, with 100% compliance, recommending breast-feeding for 4 months (scenario W) resulted in slightly more deaths than recommending breast-feeding for 6 months (scenario V) or replacement-feeding (scenario U), mortality in all 3 scenarios was very similar with a compliance of 60% or less.

#### Absolute and relative mortality rate in infants not breast-fed

**Figure 5:**
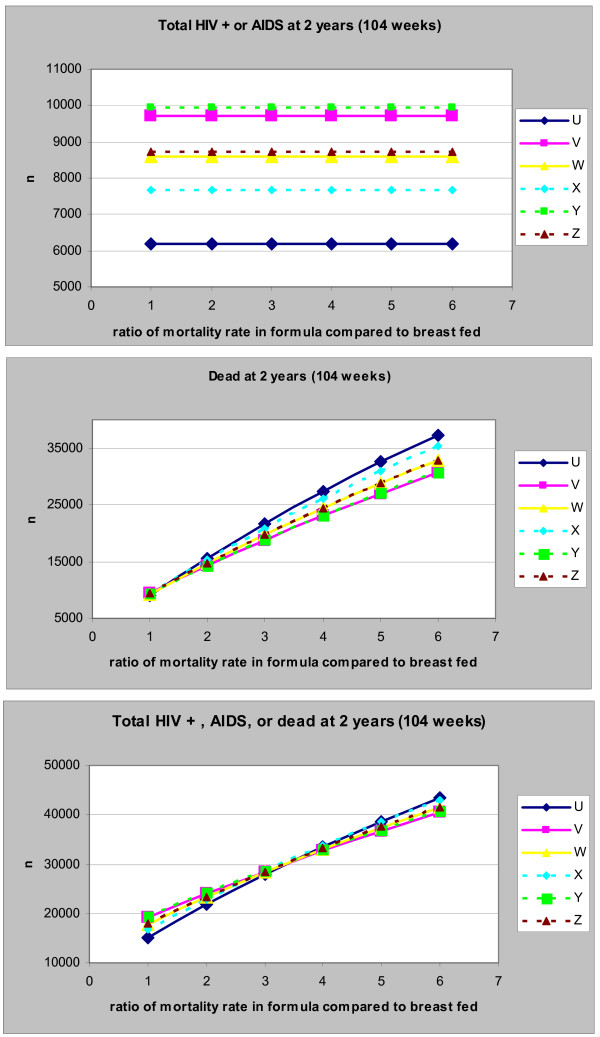
**(a-c): Impact of the relative mortality rate in replacement compared to breast-fed infants on the Number of Children with HIV/AIDS (top), dead (middle) or both (bottom graph) at 2 years**. U: Exclusive replacement-feeding (ERF) with 100% compliance; V: EBF for 6 months with 100% compliance; W: EBF for 4 months with 100% compliance: X: ERF with 70% compliance; Y: EBF for 6 months with 85% compliance; Z: EBF for 4 months with 85% compliance.

Varying the mortality rate in replacement-fed infants did not impact the total number of children with HIV/AIDS at 2 years (Additional file 1a and Figure 5a). Nevertheless increasing the absolute value of the mortality rate in replacement-fed infants (Additional file 1b) or the relative mortality in replacement-fed compared to breast-fed infants (Figure 5b) resulted in an increase in the cumulative mortality, irrespective of feeding recommendation. The increase was highest in replacement-fed infants. Although with a mortality rate less than 50 per 1000 per year, the cumulative mortality was lowest when replacement-feeding was recommended, the cumulative mortality in this scenario became highest when the mortality rate surpassed 50 per 1000 per year. Considering the total number of children with HIV/AIDS or dead at 2 years as the outcome (Additional file 1c), an equilibrium between all six scenarios was reached at a higher mortality rate: though recommending replacement feeding resulted in the lowest number of children with HIV/AIDS or dead when the mortality rate in replacement-fed was less than 150 per 1000 per year, it resulted in the highest number of children with HIV/AIDS when the mortality rate in replacement-fed was higher than 150 per 1000 per year. In terms of relative mortality, the equilibrium was reached when the mortality rate in replacement-fed infants was 3–4 fold that in breast-fed infants: recommending replacement-feeding results in the least number of children with HIV/AIDS or dead when the mortality rate in replacement-fed infants is less than 3 fold that in breast-fed infants, while the same recommendation results in the most number of children with HIV/AIDS or dead when the mortality rate in replacement-fed is more than 4 times that in breast-fed infants.

#### Rate of infection in breast-fed infants

Increasing the infection rate in breast-fed infants resulted in a higher number of children with HIV/AIDS and a higher cumulative death at 2 years in all breast-feeding scenarios (Additional file 2c). These numbers (of infected or dead children at 2 years) were even higher with a longer duration of breast-feeding. The cumulative mortality in breast-fed infants was however similar to that of replacement-fed infants when the rate of infection was in the order of 2 per 1000 per week.

#### Rate of progression from HIV to AIDS in infants

Though varying the HIV to AIDS progression rate did not impact the total number of children with HIV/AIDS or dead at 2 years, increasing the rate resulted in an exponential decrease (with little change beyond a rate of 30 per 1000 per week) in the number of children with HIV/AIDS at 2 years (Additional file 3a). Concurrently, increasing the rate resulted in an increase in the number of children dead at 2 years (Additional file 3b). The rate of increase was however lowest when replacement-feeding was recommended. Thus, although the mortality in replacement-fed infants was highest with low progression rates, recommending replacement-feeding with a high compliance resulted in the least deaths when the progression rate was higher than 20 per 1000 per week.

#### Mortality rate due to HIV/AIDS in infants

Varying the HIV/AIDS mortality rate did not affect the total number of children with HIV/AIDS or dead at 2 years (Additional file 4c). Nevertheless, increasing the rate resulted in a decrease in the number of children with HIV/AIDS at 2 years (Additional file 4a), and an increase in the number of children dead at 2 years (Additional file 4b). Despite resulting in the highest number of deaths at 2 years when HIV/AIDS mortality was low, replacement-fed infants had the least number of deaths at 2 years when HIV/AIDS mortality rate was higher than 300 per 1000 per year (Additional file 4b).

## Discussion

Policy makers and care-providers in resource-limited, high HIV-prevalence settings continue to be confronted with the dilemma of what feeding method and duration to recommend for infants born to HIV-infected children. In order to reflect conditions specific to these settings, we used epidemiologic parameters only from studies conducted in sub-Saharan studies to analyze the potential impact of 3 infant-feeding recommendations on the morbidity and/or mortality in these infants. Our analysis suggests that the choice of preferred infant-feeding method depends on the policy makers' objective: to minimize the number of children with HIV/AIDS, to minimize the cumulative mortality, or to minimize the total number of children with HIV/AIDS or dead. Most previous discussions of this issue have focused on infant mortality. This view assumes that communities will prefer minimizing deaths irrespective of the number of children that end up living with HIV. However, there is no evidence supporting this. It is our view that not only should the mortality be considered but also the number of children affected by HIV/AIDS either separately or combined with the total number of deaths. Thus an important prerequisite in the choice of feeding method should be a definition of each society's preferences and or perceptions towards mortality versus living with HIV/AIDS.

As expected, recommending breast-feeding increased the number of children infected with HIV while recommending replacement-feeding increased infant mortality. However there was only a minimal decrease in cumulative mortality when breast-feeding was recommended. Breast-feeding seemed to simply delay the timing of death rather than reduce it altogether: while breast-feeding reduced mortality at the very young ages, infants got infected and, consistent with the conditions existing in most resource limited settings, these infants progressed to AIDS relatively rapidly, and died later on by the age of two. Breast-feeding for a shorter duration (4 months as has been suggested) actually increased mortality, an increase that was accentuated when there was poor compliance. In fact, breast-feeding resulted in the least cumulative mortality only when it was recommended for six months and there was 100% (very high) compliance. Poor compliance could be expected to result in mixed-feeding with its consequences: higher infection rates, more infants who are HIV positive and who die later on. If the aim was solely to reduce mortality, then recommending breast-feeding (including breast-feeding for shorter durations) could only be justified when mortality in replacement-fed infants was greater than 50 per 1000 per year, the rate of infection in exclusively breast-fed infants was less than 2 per 1000 breast-fed infants per week, the rate of progression from HIV to AIDS was less than 15 per 1000 infected infants per week, or the mortality due to HIV/AIDS was less than 200 per 1000 infants with HIV/AIDS per year.

Reducing the recommended duration of breast-feeding resulted in fewer children living with HIV/AIDS. However, recommending replacement-feeding (even with poor compliance) resulted in the least number of children living with HIV/AIDS. This suggests that if the aim was solely to reduce the number of children living with HIV/AIDS then replacement-feeding would be the optimal choice irrespective of mortality and rates of HIV transmission and progression.

Considering the minimization of the total number of children with HIV/AIDS or dead as main objective, replacement-feeding was the best option in nearly all scenarios, the exception being when the mortality in replacement-fed infants was greater than 150 per 1000 infants or when the mortality rate in replacement-fed infants was more than 3.5 fold that in breast-fed infants.

The absence of any reduction in mortality with shorter durations of breast-feeding in this simulation supports WHO's recent update of its guidelines to recommend exclusive breast-feeding for *six *months unless replacement-feeding is AFASS. It may however be difficult for policy-makers, attempting to implement WHO guidelines, to determine the extent to which replacement-feeding in their settings is AFASS. Our simulations suggest that replacement-feeding may not be considered safe unless the mortality rate in replacement-fed infants is less than three times that in exclusively breast-fed infants.

The deterministic nature of this simulation may be a limitation because of its inherent assumption that the parameters and outcomes were fixed (having no variance). Furthermore, as for every mathematical simulation, the conclusions from our analysis could be limited by the veracity of the model selected as well as the limitations of the studies from which parameters were extracted. Our analysis is however strengthened by its particular usefulness to sub-Saharan countries as we used parameters specific to conditions in this high-HIV prevalence setting. Although the parameters used in the baseline model may differ between published studies and may be biased because of defects in the original studies, the impact of varying them was addressed in the sensitivity analysis. This sensitivity analysis of model assumptions showed our findings to be robust within the range of plausible parameters. Furthermore our findings are consistent with a recently published report by Becquet et al. who did not find any significant difference in the 2-year cumulative mortality of exclusively breast-fed infants and replacement-fed infants in West Africa[24].

## Conclusion

In conclusion, this analysis presents a framework to assist decision-makers in resource-limited settings in the choice of which infant-feeding method to recommend for infants born to HIV-infected mothers. Recommending exclusive breast-feeding in infants born to HIV-infected women in these settings, instead of replacement-feeding, may potentially result in very little gains in mortality. Furthermore, although recommending shorter durations of breast-feeding may substantially reduce infant HIV infection, it might slightly increase mortality. When exclusive breast-feeding for shorter durations is recommended, lower mortality could be achieved by a simultaneous reduction in of the rate of progression from HIV to AIDS and or HIV/AIDS mortality, reductions that are obtainable by the use of HAART in infants. Making HAART and better care available to infected-infants should thus be an imperative whenever a community and/or policy-maker prefer exclusive breast-feeding over replacement-feeding.

## Abbreviations

AFASS: Acceptable; Feasible, Affordable, Sustainable And Safe, AIDS: Acquired Immune-Deficiency Syndrome; ART: Antiretroviral Therapy; EBF: Exclusive Breast-Feeding; ERF: Exclusive Replacement-Feeding; HAART: Highly Active Antiretroviral Therapy; HIV: Human Immune-deficiency Virus; MTCT: Mother-To-Child Transmission; PCR: Polymerase Chain Reaction; USA: United States of America; WHO: World Health Organization

## Competing interests

The authors declare that they have no competing interests.

## Authors' contributions

JA conceived the study question, designed the study, collected study parameters, conducted the analysis and participated in writing the manuscript. LK designed the study, collected study parameters, conducted the analysis and participated in writing the manuscript. VS designed the study, collected study parameters and reviewed the manuscript. EES designed the study, collected study parameters and participated in writing the manuscript. All authors read and approved the final manuscript.

## Pre-publication history

The pre-publication history for this paper can be accessed here:



## Supplementary Material

Additional file 1 Impact of mortality rate in replacement-fed infants on the Number of Children with HIV/AIDS (top graph), dead (middle graph) or both (bottom graph) at 2 years. U: Exclusive replacement-feeding (ERF) with 100% compliance; V: EBF for 6 months with 100% compliance; W: EBF for 4 months with 100% compliance: X: ERF with 70% compliance; Y: EBF for 6 months with 85% compliance; Z: EBF for 4 months with 85% compliance. Click here for file

Additional file 2 Impact of the rate of infection in breast-fed infants on the Number of Children with HIV/AIDS (top), dead (middle) or both (bottom graph) at 2 years. U: Exclusive replacement-feeding (ERF) with 100% compliance; V: EBF for 6 months with 100% compliance; W: EBF for 4 months with 100% compliance: X: ERF with 70% compliance; Y: EBF for 6 months with 85% compliance; Z: EBF for 4 months with 85% compliance. Click here for file

Additional file 3 Impact of the rate of progression from HIV to AIDS in infants on the Number of Children with HIV/AIDS (top), dead (middle) or both (bottom graph) at 2 years. U: Exclusive replacement-feeding (ERF) with 100% compliance; V: EBF for 6 months with 100% compliance; W: EBF for 4 months with 100% compliance: X: ERF with 70% compliance; Y: EBF for 6 months with 85% compliance; Z: EBF for 4 months with 85% compliance. Click here for file

Additional file 4 Impact of the mortality rate due to HIV/AIDS in infants on the Number of Children with HIV/AIDS (top), dead (middle) or both (bottom graph) at 2 years. U: Exclusive replacement-feeding (ERF) with 100% compliance; V: EBF for 6 months with 100% compliance; W: EBF for 4 months with 100% compliance: X: ERF with 70% compliance; Y: EBF for 6 months with 85% compliance; Z: EBF for 4 months with 85% compliance. Click here for file
