# Supplementary material for: Potential impact of infant feeding recommendations on mortality and HIV-infection in children born to HIV-infected mothers in Africa: a simulation
Source: BMC Infect Dis. 2008 May 16;8:66. doi: 10.1186/1471-2334-8-66 (PMC2396166; doi:10.1186/1471-2334-8-66)
Supplement: Supplementary file 1 [file 1471-2334-8-66-S1.doc]

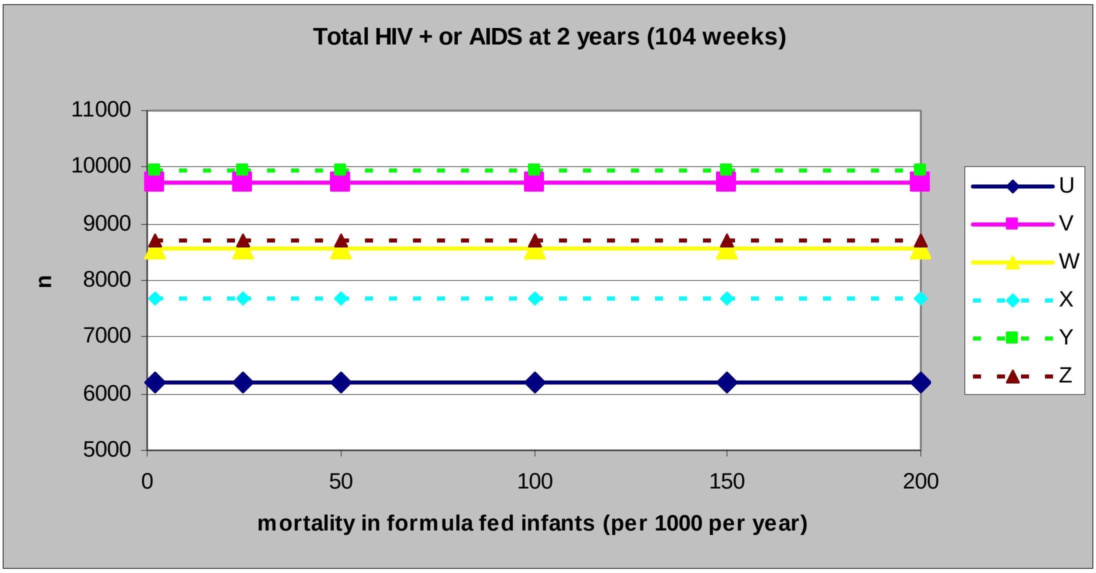
Total HIV + or AIDS at 2 years (104 weeks)
11000
10000
9000
8000
7000
6000
5000
n
0
50
100
150
200
mortality in formula fed infants (per 1000 per year)
U
V
W
X
Y
Z

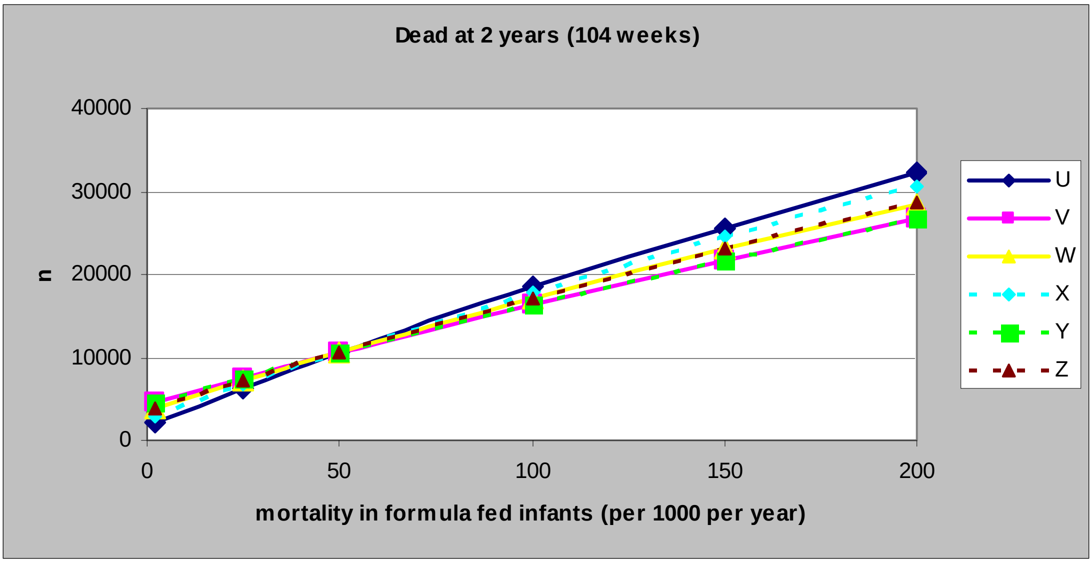
Dead at 2 years (104 weeks)
40000
30000
20000
10000
0
n
0
50
100
150
200
mortality in formula fed infants (per 1000 per year)
U
V
W
X
Y
Z

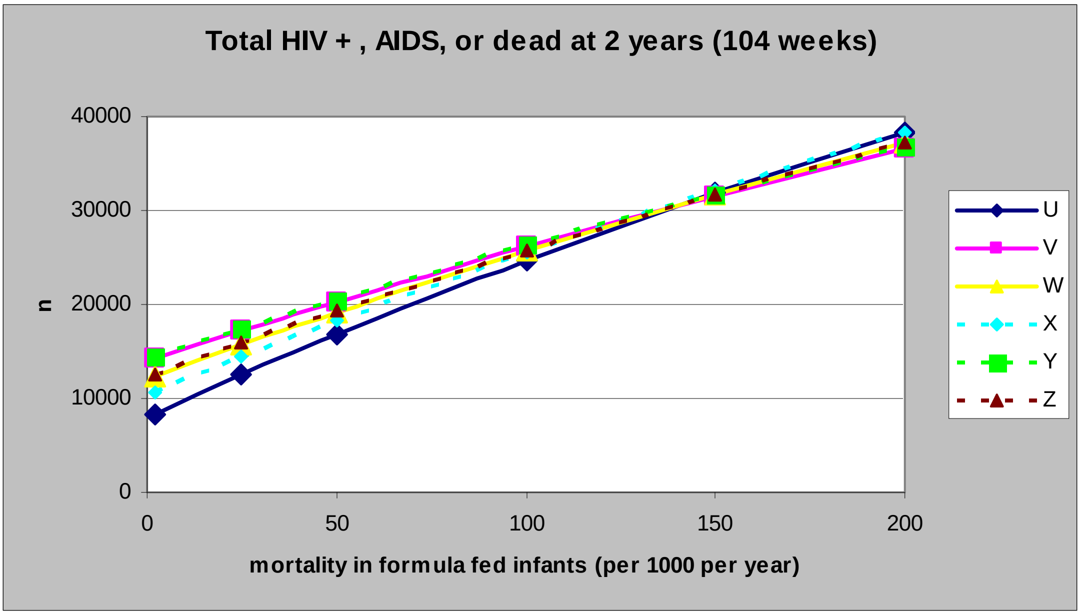
Total HIV + , AIDS, or dead at 2 years (104 weeks)
40000
30000
20000
10000
0
n
0
50
100
150
200
mortality in formula fed infants (per 1000 per year)
U
V
W
X
Y
Z
