# Supplementary material for: Potential impact of infant feeding recommendations on mortality and HIV-infection in children born to HIV-infected mothers in Africa: a simulation
Source: BMC Infect Dis. 2008 May 16;8:66. doi: 10.1186/1471-2334-8-66 (PMC2396166; doi:10.1186/1471-2334-8-66)
Supplement: Supplementary file 2 [file 1471-2334-8-66-S2.doc]

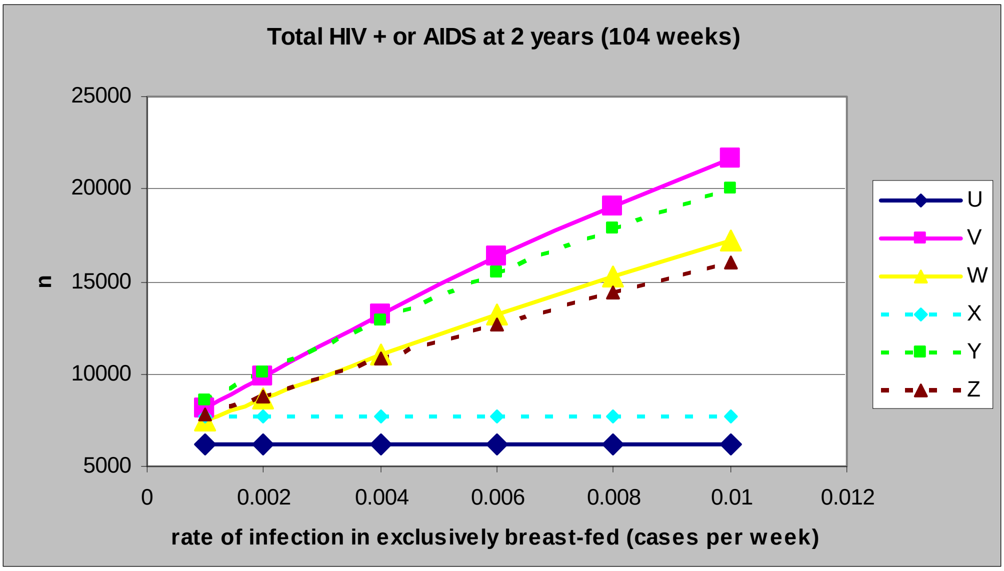

Total HIV + or AIDS at 2 years (104 weeks)
25000
20000
15000
10000
5000
n
0
0.002
0.004
0.006
0.008
0.01
0.012
rate of infection in exclusively breast-fed (cases per week)
U
V
W
X
Y
Z

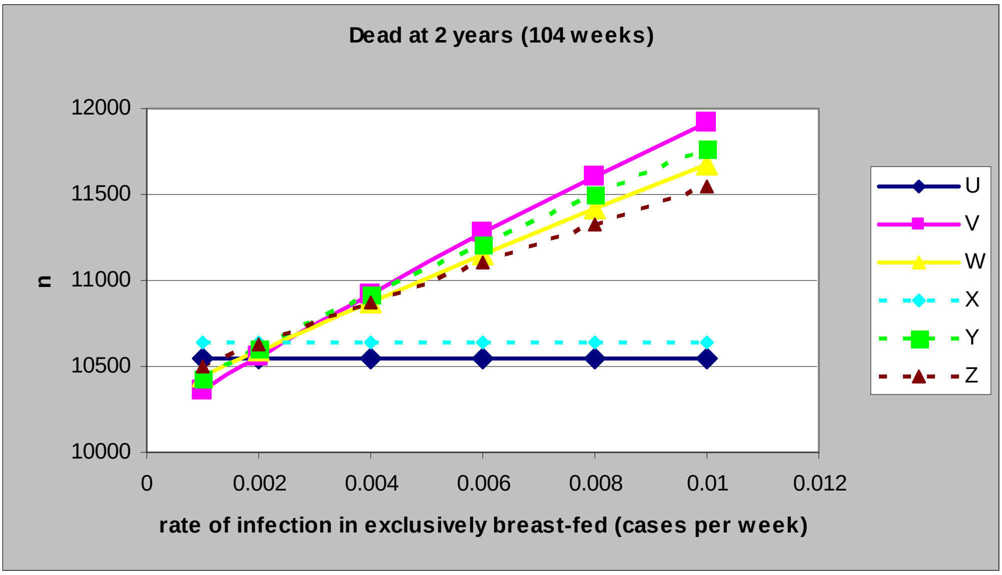

Dead at 2 years (104 weeks)
12000
11500
11000
10500
10000
n
0
0.002
0.004
0.006
0.008
0.01
0.012
rate of infection in exclusively breast-fed (cases per week)
U
V
W
X
Y
Z

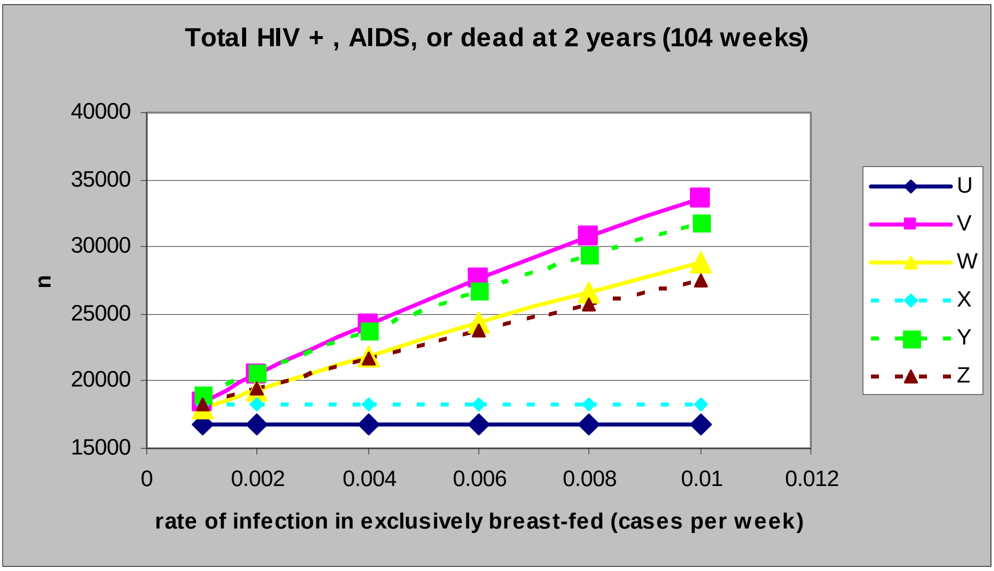

Total HIV + , AIDS, or dead at 2 years (104 weeks)
40000
35000
30000
25000
20000
15000
n
0
0.002
0.004
0.006
0.008
0.01
0.012
rate of infection in exclusively breast-fed (cases per week)
U
V
W
X
Y
Z
