# Supplementary material for: Potential impact of infant feeding recommendations on mortality and HIV-infection in children born to HIV-infected mothers in Africa: a simulation
Source: BMC Infect Dis. 2008 May 16;8:66. doi: 10.1186/1471-2334-8-66 (PMC2396166; doi:10.1186/1471-2334-8-66)
Supplement: Supplementary file 4 [file 1471-2334-8-66-S4.doc]

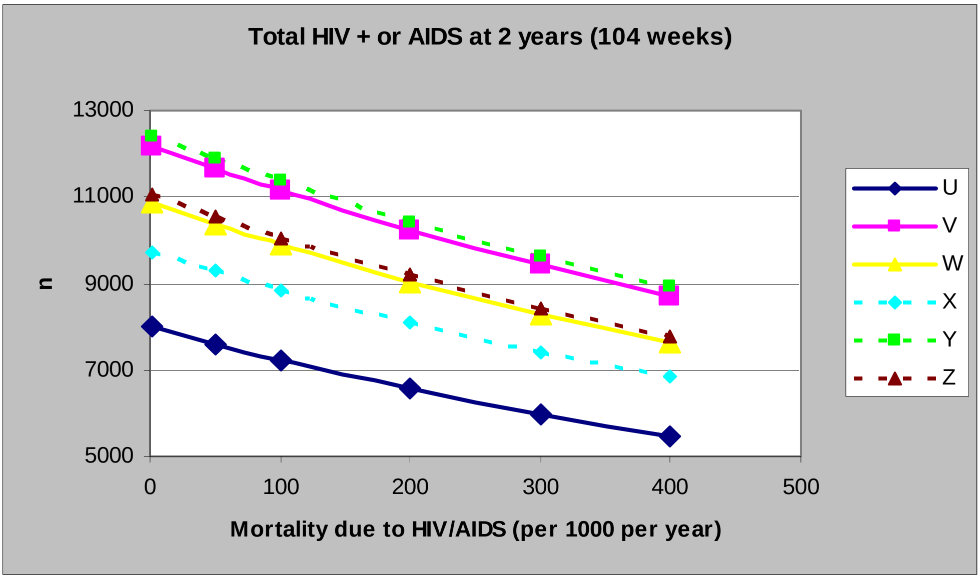
Total HIV + or AIDS at 2 years (104 weeks)
13000
11000
9000
7000
5000
n
0
100
200
300
400
500
Mortality due to HIV/AIDS (per 1000 per year)
U
V
W
X
Y
Z

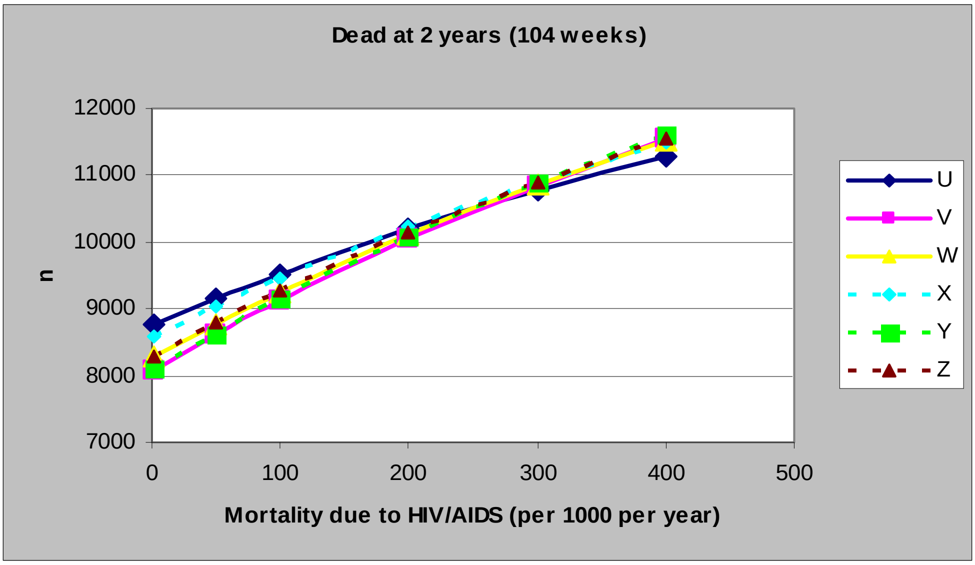
Dead at 2 years (104 weeks)
12000
11000
10000
9000
8000
7000
n
0
100
200
300
400
500
Mortality due to HIV/AIDS (per 1000 per year)
U
V
W
X
Y
Z

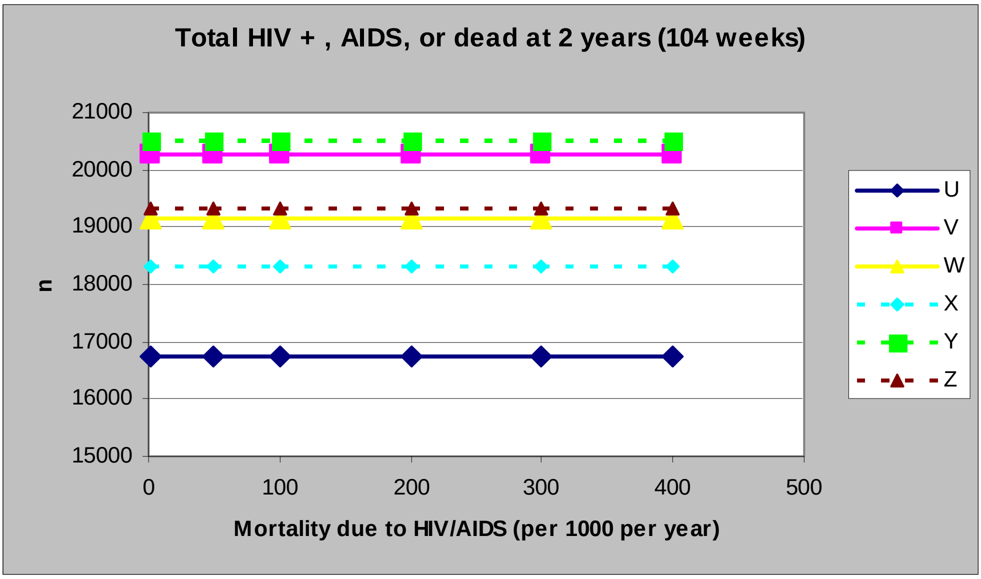
Total HIV + , AIDS, or dead at 2 years (104 weeks)
21000
20000
19000
18000
17000
16000
15000
n
0
100
200
300
400
500
Mortality due to HIV/AIDS (per 1000 per year)
U
V
W
X
Y
Z
